# Development of restrictive eating disorders in children and adolescents with long-COVID-associated smell and taste dysfunction

**DOI:** 10.3389/fped.2022.1022669

**Published:** 2022-11-24

**Authors:** Maire Brasseler, Anne Schönecker, Mathis Steindor, Adela Della Marina, Nora Bruns, Burcin Dogan, Ursula Felderhoff-Müser, Johannes Hebebrand, Christian Dohna-Schwake, Sarah C. Goretzki

**Affiliations:** ^1^ Department of Pediatrics I, Neonatology, Pediatric Intensive Care, Pediatric Infectiology, Pediatric Neurology, University of Duisburg-Essen, Essen, Germany; ^2^ Center for Translational Neuro- and Behavioral Sciences C-TNBS, University Duisburg-Essen, Essen, Germany; ^3^ West German Centre for Infectious Diseases (WZI), University Hospital Essen, University Duisburg-Essen, Essen, Germany; ^4^ Department of Pediatrics III, Pediatric Pulmonology and Sleep Medicine, University of Duisburg-Essen, Essen, Germany; ^5^ Department for Psychiatry, Psychosomatic Medicine and Psychotherapy of Children and Adolescents, LVR, Essen, Germany

**Keywords:** pediatric long COVID, anosmia, ageusia, eating disorder, anorexia

## Abstract

**Background:**

Absent or abnormal senses of smell and taste have been frequently reported during both acute and long COVID in adult patients. In contrast, pediatric patients who test positive for SARS-CoV-2 are often asymptomatic and the loss of smell and/or taste has been infrequently reported. After observing several young patients with COVID-associated anosmia and ageusia at our clinic, we decided to investigate the incidence of subsequent eating disorders in these patients and in SARS-CoV-2 positive patients who did not experience anosmia and ageusia during the same period.

**Material and methods:**

A single-site retrospective cohort study of 84 pediatric patients with suspected long COVID who were treated in the Pediatric Infectious Diseases Outpatient Clinic at the University Hospital Essen were evaluated for persistent symptoms of COVID-19. Smell and taste dysfunction as well as eating behaviors were among the signs and symptoms analyzed in this study.

**Results:**

24 out of 84 children and adolescents described smell and taste dysfunction after confirmed or suspected SARS-CoV-2 infections. A large number of these patients (6 out of 24) demonstrated increased fixation on their eating behavior post-COVID and over time these patients developed anorexia nervosa.

**Discussion/Conclusion:**

In this study we saw a possible association of long-lasting post-COVID smell and taste dysfunction with subsequent development of eating disorders. This observation is worrisome and merits further investigation by healthcare providers at multiple clinical sites.

## Introduction

Since the first patient with SARS-CoV-2 pneumonia was identified in Wuhan China in December 2019, significant morbidity and mortality associated with both the acute and long-term effects of COVID in adults has been reported worldwide. However, in children and adolescents, the majority of SARS-CoV-2-PCR-positive patients remain asymptomatic. Only a few pediatric patients present with severe acute COVID-19 (1). Apart from the acute disease, different courses of illnesses associated with previous SARS-CoV-2 infection have been reported such as Pediatric-Inflammatory-Multisystem-Syndrome temporally associated with SARS-CoV-2 (PIMS) (2–5) or long COVID and “Post-COVID-Syndrome” (6, 7).

While the two latter diagnoses are clearly defined for adults (6, 7), no clear case definition for pediatric patients exists, although children and adolescents display similar symptoms such as fatigue, muscle pain, headaches, concentration difficulty, and/or olfactory and gustatory dysfunction (8, 9). In adults, symptoms of long COVID, especially persistent olfactory dysfunction, are associated with a lower quality of life and a negative impact on mental health (10, 11). The prevalence of childhood long COVID remains unclear, as very few studies exist and among them even fewer that include a control group (10).

Moreover, the COVID pandemic has caused an increase in mental stress in children, adolescents, and their families worldwide. A rise in depression and anxiety disorders has been reported, as reflected by increased admissions due to severe eating disorders and suicide attempts (5,12–15).

During the treatment course of children and adolescents with suspected long COVID, we observed a possible association between changes in smell and taste and restrictive eating behavior. Recently, other studies have also described significantly disturbed eating habits in children with changes in smell and taste due to Long COVID (16). Several reports have revealed an increased incidence of anorexia nervosa after the onset of COVID-19 and associated measures to control the epidemic (17, 18). Hence, we retrospectively investigated smell and taste dysfunction (SAT) in our cohort to determine whether such a dysfunction represented a symptom of long COVID and whether SAT led to the development of anorexia nervosa or another eating disorder-associated disease.

## Material and methods

### Study design

Our investigation consisted of a single-site, retrospective cohort study of patients treated in the Pediatric Infectious Diseases Outpatient Clinic at the Department of Pediatrics I, University Hospital Essen. Data were collected from existing patient records.

### Ethics approval

This study was approved by the Ethics Committee of the Medical Faculty of the University Duisburg-Essen (22-10581-BO) and conducted in accordance with the latest version of the Declaration of Helsinki. As the study presents a retrospective analysis with anonymous data, the ethics committee waived the need for informed consent.

### Eligibility criteria

Included were all patients (age 0–18 years, *n* = 84) treated in the Pediatric Infectious Diseases Outpatient Clinic at the Department of Pediatrics I, University Hospital Essen from April 2021 to April 2022 with a diagnosis of suspected long COVID. Twenty-four patients, who met the criteria of long COVID according to the German Association of the Scientific Medical Societies (AWMF) and NICE guidelines (6, 7) and reported changes in their olfactory and/or gustatory senses, were further evaluated. These patients were divided into two groups according to the presence (“LC+SAT+ED”-group) or absence (“LC+SAT”-group) of restrictive eating behavior and anorexia nervosa as defined by the International Classification of Disease ICD-11 (19). Patients without changes in their eating behavior and no diagnosed eating disorder comprised the “LC+SAT”-group (see Diagram 1).

### Exclusion criteria

In cases where the diagnosis of long COVID could not be confirmed (*n* = 25), SAT was not reported (*n* = 30), or the long COVID diagnosis was rejected due to insufficient data (*n* = 5), patients were excluded.

### Diagnostic workup

A detailed medical history, blood tests (see Supplementary material), physical examination, oxygen saturation, blood pressure, and a consultation with an Ear Nose and Throat (ENT) specialist were obtained from all patients. Body mass indices (BMI; kg/m²) were assessed using BMI-centiles adapted to age, sex, and race (20). All further consultations with other medical specialists were conducted based on clinical presentation. Electroencephalogram (EEG), electrocardiogram (ECG), electrocardiography (ECHO), ultrasound of the abdomen and thyroid, chest-x-rays, lung function with lung clearance index (LCI) measurement, cranial or spinal magnetic resonance imaging (MRI), and Nerve Conduction Velocity (NCV) were performed if necessary. We recommended a consultation with a child and adolescent psychiatrist for all patients with newly developed restrictive eating behavior to rule out or confirm the diagnosis of a possible eating disorder and initiate treatment. Demographic variables, preexisting diagnoses, and newly detected underlying diseases as well as other known long COVID-associated symptoms (fatigue, headaches, etc.) were documented. Patients were systematically queried as to symptoms associated with long COVID and restrictive eating behavior and psychological or other somatic symptoms were included in the evaluation. Amenorrhea was defined by three or more missing menstrual cycles; menstruation was defined as irregular if a time shift of more than 30% had occurred for three months after at least three months of regular menstrual cycles. The subjective change in recalled symptoms over time was monitored. An improvement was defined by less reported intensity, quantity of symptoms, and/or alleviation of restrictions in daily routines, and numbers of symptoms or less restrictions in daily routines caused by long COVID symptoms.

### Statistics

Statistical analysis was performed with SPSS 27 Statistic Software (IBM; Armonk, NY). Continuous variables were presented as median. The confidence interval (CI) was set at 95%. Mann-Whitney-*U*-test for continuous variables or Chi-Square test for categorical variables were used to test for differences between cases and control groups. The level of significance was set at *p* = 0.05. Percentiles for height, weight, and BMI were used according to ICD-11, 6B80 Anorexia Nervosa.

## Results

Twenty-four (28.6%) of the 84 patients evaluated in this study met both the criteria of long COVID according to the AWMF Guidelines (see Table 1) and had SAT. These symptoms were evaluated by systematic anamnesis and excluded anatomic reasons (Table 2 and Diagram 1 in Supplementary material). Six patients (3 male and 3 female) showed restrictive eating behavior and 5 of them met the ICD-11 guidelines for anorexia nervosa (25% of the children with SAT and 8.6% of the entire group with suspected long COVID) (see Diagram 1). None of the long COVID patients without SAT showed differences in their eating behavior or developed anorexia nervosa.

**Table 1:** Definitions.

Definition	
AWMF Long COVID	– Persisting symptoms after acute COVID-19 or its treatment – Symptoms leading to a new health impairment – New symptoms that appeared after the end of the acute phase as a result of COVID-19 disease – Worsening of a pre-existing underlying condition (6)
NICE Long COVID	– Ongoing symptomatic signs and symptoms that developed during COVID-19 and have no other explanation from 4 weeks up to 12 weeks after the acute infection – >12 weeks = post-COVID-19 syndrome. It usually presents with clusters of symptoms, often overlapping, which can fluctuate and change over time and can affect any system in the body. (7)
Definition used for changes in olfactory senses	– Decreased taste and/or smell (Hyposmia/Hypogeusia) – Loss of taste and/or smell (Anosmia/Ageusia) – Unpleasant smell and/or taste, commonly a rancid odor/flavor
ICD-11 Definition Anorexia nervosa	– Significantly low body weight for the individual's height, age, developmental stage, or weight history (body mass index [BMI)] < 18.5 kg/m^2^ in adults and under 5th percentile in children and adolescents; rapid weight loss [e.g., > than 20% of total body weight within 6 months]) – Low body weight not better accounted for by another medical condition or the unavailability of food – Persistent pattern of restrictive eating or other behavior aimed at establishing or maintaining abnormally low body weight, typically associated with extreme fear of weight gain – Excessive preoccupation with body weight or image (15)

**Table 2:** Diagnostics and therapy.

	LC + SAT + ED (*n* = 6)		LC + SAT (*n* = 18)	
Diagnostic Procedures	Pathological: Echocardiography: 1 MRI: 1 (cranial, post mastoiditis)	Normal: lung function: 6 ECG: 4 Echocardiography: 2 Chest x-ray: 3 MRI: 3 planned NCV: 1 (1 planned) EEG: 5 ultrasound:	Pathological: lung function: 4 ECG: 1 Echocardiography: 1 EEG: 2 ultrasound: 1 NCV: 1 chest x-ray: 1	Normal: lung function: 12 ECG: 13 Echocardiography: 14 chest x-ray: 6 MRI: 3 NCV: 2 EEG: 4 ultrasound: 5
Consultants	Pathological: Child and adolescence psychologist: 5	Normal: NCV: 5 Neuropediatric: 4 Ophthalmology: 3	Pathological: Pneumology: 3 Neuropediatric: 1 Ophthalmology: 1	Normal: Neuropediatric: 4 Ophthalmology: 1 Pneumology: 1 Cardiology: 1 Gastroenterology: 1
Therapy	Smell training: 5 Psychological consultation: 4 Mother child rehabilitation: 1 Symptom diary: 2		Smell training: 16 Psychological consultation: 2 Physiotherapy: 2 Mother child rehabilitation: 1 Symptom diary: 2 Lifestyle changes: 2	

The 24 patients were ages 6–17 years. SARS-CoV-2 infection had been confirmed by PCR tests in all patients. None of the 24 patients with SAT had been hospitalized due to COVID-19. None of them suffered from gastrointestinal symptoms during acute or long COVID. Long COVID symptoms without an association to eating disorders included: decreased general condition (87.5%), “brain fog”/concentration difficulty (37.5%), hyperesthesia (50.0%), and headaches (75.0%). There were no significant differences between concentrations of SARS-CoV-2 antibodies in the “LC + SAT + ED”-group and “LC + SAT”-group (median: 125.6 BAU/ml [binding antibody units/mL] vs. 450 BAU/ml; standard deviation 870.8). Additionally, there were no significant differences in SARS-CoV-2 viral variants between the two groups (see Table 3).

**Table 3:** Patient characteristics and COVID-19 associated symptoms.

	*LC+SAT+ED* (*n* = 6)	*LC+SAT* (*n* = 18)
Age (years)	8–14 (Median 11.5)	6–17 (Median 14)
Sex	3 female, 3 male	13 female, 5 male
Numbers of MD consultations since the first signs of long COVID before presentation at our Pediatric Infectious Diseases Outpatient Clinic	1–5 (Median 2.5)	1–5 (Median 3)
	Fever: 4 (66.7%)	Fever: 4 (22.2%)
	Flu-like symptoms: 3 (50.0%)	Flu-like symptoms: 13 (72.2%)
	Chest pain: 2 (33.3%)	Chest pain: 1 (5.6%)
	Headache: 5 (83.3%)	Headache: 11 (61.1%)
Initial symptoms during Covid 19	Smell and taste dysfunction: 1 (16.7%)	Smell and taste dysfunction: 1 (5.6%)
	Pain in Extremities: 1 (16.7%)	Pain in Extremities: 1 (5.6%)
	Vertigo: 2 (33.3%)	Vertigo: 9 (50.0%)
	Diarrhea: 0 (0.0%)	Diarrhea: 1 (5.6%)
	Unknown: 1	Unknown: 10
Subtype of SARS-CoV2 (PCR)	Delta/Indian B.1.617.2: 4	Delta/Indian B.1.617.2: 5
	Alpha/British B.1.1.7: 0	Alpha/British B.1.1.7: 1
	Omicron B.1.1.529: 2	Omicron B.1.1.529: 2
SARS-CoV2-antibodies (BAU/ml)	2020–0 (Median: 126)	>2090–0 (Median: 322)
Max. Loss of weight after COVID-19 (kilogram)	4–6.5 (Median: 5)	1 (No median, only 1 patient)
	95%-CI: 4.33–6.17	95%-CI: 0.0–0.17
	Before long COVID:	Before long COVID:
	18.1–23.1	15.5–25.7
	95%-CI: 17.7–22.1	95%-CI: 18.1–21.8
	Actual BMI:	Actual BMI:
Actual body mass index (and before long COVID) (kg/m^2^)	14.8-18.4	15.5-25.7
	95%-CI: 15.4–18.3	95%-CI: 18.2–22.1
	BMI reduction:	BMI reduction:
	95%-CI: 1.77-5.58	95%-CI: 0.0–0.18
Days until the symptoms improved	0–84	0–120 days

Five of the 6 patients with restrictive eating behavior reported a reduced appetite. This symptom, as well as hair loss or changes in menstrual cycle were significantly more common in the “LC+SAT+ED”-group than in the “LC+SAT”-group (hair loss *p* = 0.001, menstrual cycle *p* = 0.042, Chi-quadrant). In addition, 5 patients reported symptoms of depression, stress, and no longer enjoying food. Noticeably all patients felt misunderstood by family, friends, and professionals. Depressive symptoms and anxiety were significantly more common in the “LC+SAT+ED”-group than in the “LC+SAT”-group (*p* = 0,005, Mann-Whitney-*U*-test, *z* = −2,835) (see Table 4); one of the “LC+SAT”-group patients had been diagnosed with depression and an eating disorder before COVID infection, which did not worsen during the course of disease.

**Table 4:** Long COVID symptoms and eating behavior.

	*LC+SAT+ED* (*n* = 6)	*LC+SAT* (*n* = 18)
Long-lasting GI symptoms	Changes in quality of taste and smell: 6 (100.0%)	Changes in quality of taste and smell: 18 (100.0%)
	Reduced smell and taste: 3 (50.0%)	Reduced smelling and tasting: 9 (50.0%)
	Reduced appetite: 5 (83.3%)	Reduced appetite: 2 (11.1%)
	Abdominal pain: 0	Abdominal pain: 3 (16.7%)
	Constipation: 2 (33.3%)	Constipation: 4 (22.2%)
Somatic symptoms of eating disorder	Irregular menstrual cycle: 3 (100.0% of females)	Irregular menstrual cycle: 2 (15.4% of females)
	95%-CI: 0.0-1.07	95%-CI: 0.0-0.27
	Hair loss:	Hair loss:
	3 (50.0%), 95%-CI: 0.00–1.07	2 (11.1%), 95%-CI: 0.0-0.27
	Sleep problems: 3 (50.0%)	Sleep problems: 6 (33.3%)
	Fatigue: 3 (50.0%)	Fatigue: 6 (33.3%)
	Less condition: 5 (83.3%)	Less condition: 16 (88.9%)
	Headache: 4 (66.7%)	Headache: 14 (77.8%)
	Dyspnea: 1 (16.7%)	Dyspnea: 6 (33.3%)
Other long-lasting symptoms	Paresthesia: 2 (33.3%)	Paresthesia: 10 (55.6%)
	Palpitation: 3 (50.0%)	Palpitation: 4 (22.2%)
	Concentration problems: 4 (66.7%)	Concentration problems: 8 (44.4%)
	Chest pain: 1 (16.7%)	Chest pain: 1 (5.6%)
	Vertigo: 1 (16.7%)	Vertigo: 4 (22.2%)
Psychological symptoms	Self-reported depression symptoms: 5 (83.3%)	Self-reported depression symptoms: 5 (27.8%)
	Self-reported anxiety symptoms: 4 (66.7%)	Self-reported anxiety symptoms: 5 (27.8%)
	95%-CI: 0.40–1.26	95%-CI: 0.05–0.51
Restrictive eating behavior	Restrictive eating behavior: 6	0
	95%-CI: 1.0–1.0	95%-CI: 0.0–0.0
Permanent weight control	2 (33.3%)	0
Body Scheme	Distorted body image: 4 (66.7%)	Distorted body image: 1 (5.6%)
	Body checking: 2 (33.3%)	
Suicidal ideation	Suicidal thoughts: 1 (16.7%)	None

None of the “LC+SAT+ED”-group or “LC+SAT”-group patients followed a special diet (e.g., vegetarian, vegan, or gluten-free). The 6 patients of the “LC+SAT+ED”-group reported restrictive eating behavior and lost between 4 and 6.5 kilograms of body weight (see Tables 3, 5). The period from the beginning of long COVID symptoms to the maximum loss of weight was between 3 and 5 months. Four of the patients reported a distorted body image and two of them had to weigh themselves frequently. No compensatory mechanisms were reported. Associated symptoms like decreased general condition, amenorrhea, constipation, and malnutrition also existed (see Table 4). None of the “LC+SAT”-group patients lost weight (see Table 3). Weight loss and BMI reduction during long COVID were significantly higher in the the 6 patients of the “LC+SAT+ED”-group (*p* < 0.001, *p* = 0.009, chi-quadrant, Table 4) than in patients of the “LC+SAT”-group.

**Table 5:** Signs of eating disorder within the six described *LC+SAT+ED*

Signs of anorexia nervosa ICD 11	Patient 1	Patient 2	Patient 3	Patient 4	Patient 5	Patient 6
	BMI before LC 21.3	BMI before LC 18.2	BMI before LC 23.1	BMI before LC 20.5	BMI before LC 18.2	BMI before LC 18.1
Body mass index (BMI) < 18.5 kg/m^2^; < 5th percentile in children and adolescents; rapid weight loss (e.g., > than 20% of total body weight within 6 months)	lowest during LC: BMI 16.3	lowest during LC: BMI 16.2	lowest during LC: BMI 16.5	lowest during LC: BMI 17.4	lowest during LC: BMI 16.1	lowest during LC: BMI 14.8
	7.	8.	11.	14.	36.	5.
	Percentile in 3 months 14% decrease	Percentile in 3 months 14% decrease	Percentile in 3/4 months 21% decrease	Percentile in 3 months 21% decrease	Percentile in 4 months 16% decrease	Percentile in 5/6 months 19% decrease
Low body weight is not better accounted for by another medical condition or the unavailability of food.	No other explanations	No other explanations	No other explanations	No other explanations	No other explanations	No other explanations
A persistent pattern of restrictive eating or other behavior aimed at establishing or maintaining abnormally low body weight, typically associated with extreme fear of weight gain.	Yes	Yes	Yes	Yes	Yes	Yes
Excessive preoccupation with body weight or image.	Yes	Yes	Yes	Yes	Yes	Yes
Somatic effects of the eating disorder						
- Hair loss			X	X		X
- Changes in the menstrual cycle	X		X	X		X
- Less energy	X	X	X	X	X	

Treatment recommendations included training of the olfactory nerve (twice a day smelling of 4 different essential oils, each for a period of 10 s), keeping a diary of symptoms, weight check-ups by a pediatrician, consultations with a child and adolescent psychiatrist, and provision of emergency contacts to both patients and their families (Table 2). These recommendations were made with the intention of symptom management. In 5 of the 6 patients of the “LC+SAT+ED”-group, the diagnosis of anorexia nervosa was confirmed during further examinations by a child and adolescent psychiatrist or a psychologist. Three of the “LC+SAT+ED”-group patients reported fewer symptoms after 32 to 84 days. One of them reported decreased symptoms following SARS-CoV-2-vaccination (Pfizer/BioNTech). Three patients attributed their improvementto psychotherapy.

## Discussion

Here, we describe a group of 24 pediatric patients ages 6–17 years with long COVID or Post-COVID Syndrome and concurrent SAT. A clinically important proportion of patients developed a restrictive eating behavior and secondary eating disorder as defined by ICD-11. SAT is a widely recognized symptom of acute SARS-CoV-2 infection. In cases of COVID-19 persistence for more than 4 weeks, the diagnosis of long COVID was confirmed; when persistence continued beyond 12 weeks, Post-COVID Syndrome was confirmed. A relevant impact of long COVID on the mental health in affected adults has already been documented (10). However, to our knowledge, the association of SAT dysfunction and eating disorders in childhood has not been reported to date.

Even if the prevalence of long COVID in children remains unclear (10), some studies report its ubiquity in children with symptoms like persistent fatigue (25.2%), cognitive sequelae such as irritability (24.3%) and mood changes (23.3%), headaches (16.9%), rhinorrhea (16.1%), coughing (14.4%), and anosmia/dysgeusia (12.3%) (8, 9). Patients included in this study presented with these symptoms.

Within the study population all patients with a secondary eating disorder showed somatic symptoms (reduced fitness, constipation, malnutrition and amenorrhea) and were ages 8–14 years. Normally, the incidence of eating disorders is highest for girls ages 15–19 years and for boys ages 10–14 years (21). In all 6 patients in this study, the focus on food and restrictive eating occurred after quality changes in smell and taste. Increased attention to eating behavior like obsession with the daily meal plan is a risk factor for the development of eating disorders and may have promoted their development (22). In adults, persistent olfactory dysfunction has been associated with a lower quality of life and impaired mental health (10).

Female patients are usually more prone to develop an eating disorder than males. Only 5%–10% of patients who were reported with anorexia and bulimia nervosa during the COVID pandemic were male (23–26). Our results showed an equal distribution of both sexes who developed an eating disorder due to restrictive eating behavior. This implies that long COVID may affect male and female adolescents alike and override the usual gender differences in eating disorders, suggesting that SAT represents a clinically important individual risk factor for a new eating disorder.

Well documented research has proven that patients with eating disorders, especially with anorexia nervosa, are at risk of many somatic and psychiatric comorbidities and higher mortality (15, 27–30). Our study results show a positive correlation between restrictive eating behavior and symptoms of depression. These facts and the significant loss of weight in our patients emphasizes the importance of assessing both female and male pediatric patients for possible eating disorders after COVID-19. Physicians, caregivers, and patients should be able to recognize symptoms of an eating disorder, especially in all cases of SAT. Follow-up departments with multidisciplinary teams are needed for children and adolescents who experience signs of long COVID. In the case of SAT, screening methods like the SCOFF Questionnaire to detect eating disorders in children and adolescents at an early stage could be helpful (31).

There are several limitations to our study. As there is no acknowledged definition for long COVID in pediatric patients, we used the definition for adults which might not be suitable for pediatric patients in every sense. In addition, smell and taste dysfunctions are subjective symptoms. Only 5 patients were diagnosed by a mental health specialist prior to our study because of very limited resources due to higher demand for psychological and psychiatric services for children during the pandemic. Therefore, not all of our patients had the opportunity to get standard psychological evaluations during their visit in our outpatient clinic. Due to the different home addresses of patients included in this study and different geographic locations of their respective healthcare providers, the corresponding psychiatrist or psychologist was not always the same person. Furthermore, as long COVID is a relatively rare and new diagnosis, in this single-site retrospective cohort study we only report on a small patient group, which may result in statistical biases.

## Conclusion

Pediatric patients with the diagnosis of long COVID and changes in smell and taste seem to be at risk of developing an eating disorder. Pediatricians should be aware of red flags while obtaining a medical history and performing a physical examination of these patients. As stated on many occasions, the mental health of children and young adults has suffered during the pandemic of SARS-CoV-2 (13). The possibility of consultation with a Child and Adolescent Psychiatrist or a Child Psychologist and access to necessary psychotherapies are urgently needed (32). Screening methods to detect secondary eating disorders in patients suffering from long COVID could be a helpful to pediatricians and child psychiatrists. Collaboration with child and adolescent psychiatrists as well as teachers, sports coaches, social workers, and families is even more necessary during the COVID-19 pandemic than in non-COVID-19 times. More research with larger patient groups is needed to support the hypothesis that changes in taste and smell due to long COVID are a risk for developing anorexia nervosa in pediatric patients. We hope to test this in a multicenter prospective study in the near future.

## Data Availability

The original contributions presented in the study are included in the article/**Supplementary Material**, further inquiries can be directed to the corresponding author/s.
