# Supplementary material for: Development of restrictive eating disorders in children and adolescents with long-COVID-associated smell and taste dysfunction
Source: Front Pediatr. 2022 Nov 24;10:1022669. doi: 10.3389/fped.2022.1022669 (PMC9743173; doi:10.3389/fped.2022.1022669)
Supplement: Supplementary file 1 [file Presentation1.pdf]

Supplementary Material

Standard diagnostics tests performed for every patient

Blood workup: Differential blood count, TPZ, INR, aPTT, thrombin time, fibrinogen, antithrombin-III, d-dimer, electrolytes, potassium, phosphate, creatinine, cystatin-C, urea, CK, CK-MB, troponin T, myoglobin, NT-pro-BNP, transaminases, LDH, CRP, triglycerides, C3, C4, haptoglobin, IgA/M/G/E, blood sedimentation, blood gas analysis Additional tests performed for patients seen after11/2021: 25-OH-vitamin D, vitamins B1, B6, and B12, folate and sprue diagnostics, ferritin

Standard urinalysis: The laboratory diagnostics were expanded in a symptom-oriented manner.

To assess the eating behavior, the following questions were asked and records collected:

Record history of changes in the eating behavior in the past and history of defecation and diuresis.

Do you follow a special diet (for example vegetarian, vegan, gluten-free)?

Have you noticed any changes in your eating behavior?

Are there changes in the amount you are eating?

Are you joining your family or classmates for meals?

Do you like food you did not like before?

Is there any food you loved, which you do not like anymore?

Are you skipping meals?

Do you have cravings you did not have before (salt, sweets, fat, special foods)?

Are you eating at the same times as before (night eating)?

Are there changes in the form of how or where you get your main meals?

Do you feel sick after food intake of any kind?

Did you have to vomit after eating in the past?

When there have been changes in your eating behavior, did these occur before or after SARS-CoV-2 infection?

To assess smell and taste dysfunction, the following questions were asked and records collected:

Record history of sinus disorders, ENT infections, head trauma or surgery, allergies, medication use, and exposure to chemicals or fumes.

Have you noticed any changes in the way food tastes?

Does anything taste different then before?

Can you taste everything? Do you sometimes experience the taste of a food or beverage even when you haven't eaten or drunk anything?

When there have been changes did you associate them with certain food or drinks?

When there have been changes in your taste, did you experience them before, during, and/or after since your SARS-CoV-2 infection?

Do those changes bother you?

Are there any changes in your smell?

Does anything smell different then before?

Can you smell everything?

Do you notice certain odors even when other people say there isn’t one?

When there have been smell changes did you associate them with certain foods or beverages?

When there have been changes in your smell, did you experience them before, during, and/or after your SARS-CoV-2 infection?

Do those changes bother you?
Do you experience rancid odors frequently?
Have you been to an ENT doctor? Did they test and record your olfactory senses?
The above questions regarding eating behavior, smell, and taste were expanded or shortened in a symptom-oriented manner.
