# Supplementary material for: Development of restrictive eating disorders in children and adolescents with long-COVID-associated smell and taste dysfunction
Source: Front Pediatr. 2022 Nov 24;10:1022669. doi: 10.3389/fped.2022.1022669 (PMC9743173; doi:10.3389/fped.2022.1022669)
Supplement: Supplementary file 2 [file Datasheet1.doc]

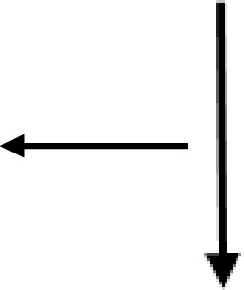

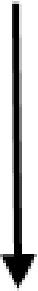


Excluded patients due to missing data n = 5

Diagram 1

Patients with referral diagnosis of Long COVID (April 2021 to April 2022)

Suspected Long COVID n = 84

Long COVID with smell and taste dysfunction n = 29


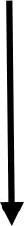


Excluded n = 55:

Long COVID criteria not met n = 25

No smell and taste dysfunction n = 30


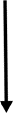


“*LC+SAT+ED*” n = 6

“*LC+SAT*” n = 23

Final “*LC+SAT”*-group n = 18

- Met criteria of long COVID
- Showed smell and taste dysfunction
- No sign of eating disorder

Final *“LC+SAT+ED”*-group n = 6

- Met criteria of long COVID
- Showed smell and taste

dysfunction

- Met criteria of restrictive disorder
