# Supplementary material for: Development of restrictive eating disorders in children and adolescents with long-COVID-associated smell and taste dysfunction
Source: Front Pediatr. 2022 Nov 24;10:1022669. doi: 10.3389/fped.2022.1022669 (PMC9743173; doi:10.3389/fped.2022.1022669)
Supplement: Supplementary file 3 [file Table4.doc]

**Table 4.** Long COVID Symptoms and Eating Behavior

|  | *LC+SAT+ED* (n = 6) | *LC+SAT* (n = 18) |
| --- | --- | --- |
| Long-lasting GI symptoms | Changes in quality of taste and smell: 6 (100.0%)  Reduced smell and taste: 3 (50.0%)  Reduced appetite: 5 (83.3%)  Abdominal pain: 0  Constipation: 2 (33.3%) | Changes in quality of taste and smell: 18 (100.0%)  Reduced smelling and tasting: 9 (50.0%)  Reduced appetite: 2 (11.1%)  Abdominal pain: 3 (16.7%)  Constipation: 4 (22.2%) |
| Somatic symptoms of eating disorder | Irregular menstrual cycle: 3 (100.0% of females)  95%-CI: 0.0-1.07  Hair loss:  3 (50.0%), 95%-CI: 0.00-1.07 | Irregular menstrual cycle: 2 (15.4% of females)  95%-CI: 0.0-0.27  Hair loss:  2 (11.1%), 95%-CI: 0.0-0.27 |
| Other long-lasting symptoms | Sleep problems: 3 (50.0%)  Fatigue: 3 (50.0%)  Less condition: 5 (83.3%)  Headache: 4 (66.7%)  Dyspnea: 1 (16.7%)  Paresthesia: 2 (33.3%)  Palpitation: 3 (50.0%)  Concentration problems: 4 (66.7%)  Chest pain: 1 (16.7%)  Vertigo: 1 (16.7%) | Sleep problems: 6 (33.3%)  Fatigue: 6 (33.3%)  Less condition: 16 (88.9%)  Headache: 14 (77.8%)  Dyspnea: 6 (33.3%)  Paresthesia: 10 (55.6%)  Palpitation: 4 (22.2%)  Concentration problems: 8 (44.4%)  Chest pain: 1 (5.6%)  Vertigo: 4 (22.2%) |
| Psychological symptoms | Self-reported depression symptoms: 5 (83.3%)  Self-reported anxiety symptoms: 4 (66.7%)  95%-CI: 0.40-1.26 | Self-reported depression symptoms: 5 (27.8%)  Self-reported anxiety symptoms: 5 (27.8%)  95%-CI: 0.05-0.51 |
| Restrictive eating behavior    Permanent weight control | Restrictive eating behavior: 6  95%-CI: 1.0-1.0  2 (33.3%) | Restrictive eating behavior: 0  95%-CI: 0.0-0.0  0 |
| Body Scheme | Distorted body image: 4 (66.7%)  Body checking: 2 (33.3%) | Distorted body image: 1 (5.6%) |
| Suicidal ideation | Suicidal thoughts: 1 (16.7%) | None |
